# Predicting therapy dropout in chronic pain management: a machine learning approach to cannabis treatment

**DOI:** 10.3389/frai.2025.1557894

**Published:** 2025-02-20

**Authors:** Anna Visibelli, Rebecca Finetti, Bianca Roncaglia, Paolo Poli, Ottavia Spiga, Annalisa Santucci

**Affiliations:** ^1^ONE-HEALTH Lab, Department of Biotechnology, Chemistry and Pharmacy, University of Siena, Siena, Italy; ^2^POLIPAIN CLINIC, SIRCA Italian Society of Cannabis Research, Pisa, Italy; ^3^Centro della Scienza e della Tecnica, Polo Universitario Grossetano, Grosseto, Italy; ^4^Competence Center, ARTES 4.0, Siena, Italy

**Keywords:** dropout, cannabis, therapy, machine learning, pain treatment, pharmacogenetics, precision medicine

## Abstract

**Introduction:**

Chronic pain affects approximately 30% of the global population, posing a significant public health challenge. Despite their widespread use, traditional pharmacological treatments, such as opioids and NSAIDs, often fail to deliver adequate, long-term relief while exposing patients to risks of addiction and adverse side effects. Given these limitations, medical cannabis has emerged as a promising therapeutic alternative with both analgesic and anti-inflammatory properties. However, its clinical efficacy is hindered by high interindividual variability in treatment response and elevated dropout rates.

**Methods:**

A comprehensive dataset integrating genetic, clinical, and pharmacological information was compiled from 542 Caucasian patients undergoing cannabis-based treatment for chronic pain. A machine learning (ML) model was developed and validated to predict therapy dropout. To identify the most influential factors driving dropout, SHapley Additive exPlanations (SHAP) analysis was performed.

**Results:**

The random forest classifier demonstrated robust performance, achieving a mean accuracy of 80% and a maximum of 86%, with an AUC of 0.86. SHAP analysis revealed that high final VAS scores and elevated THC dosages were the most significant predictors of dropout, both strongly correlated with an increased likelihood of discontinuation. In contrast, baseline therapeutic benefits, CBD dosages, and the CC genotype of the rs1049353 polymorphism in the CNR1 gene were associated with improved adherence.

**Discussion:**

Our findings highlight the potential of ML and pharmacogenetics to personalize cannabis-based therapies, improving adherence and enabling more precise management of chronic pain. This research paves the way for the development of tailored therapeutic strategies that maximize the benefits of medical cannabis while minimizing its side effects.

## 1 Introduction

**Figure 1:**
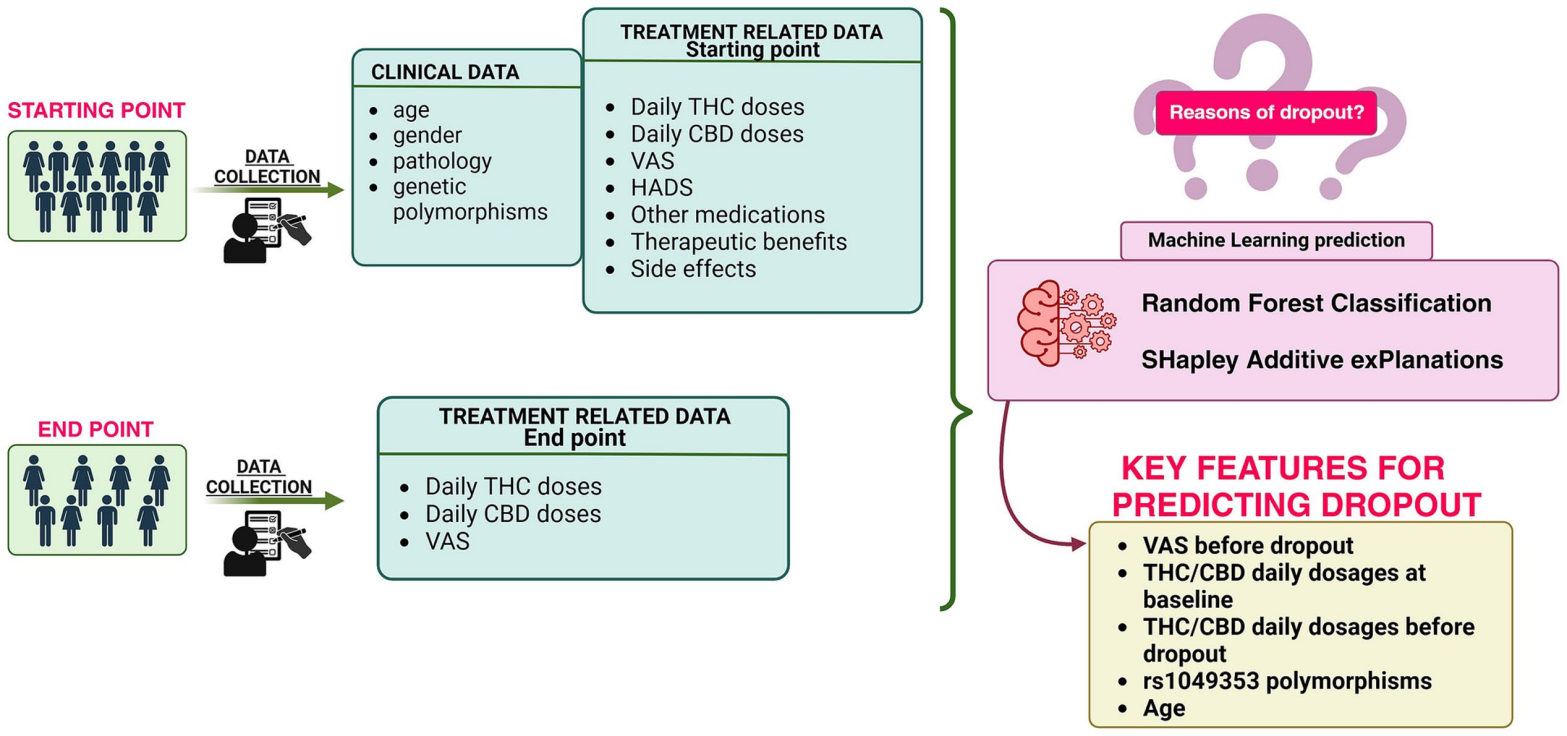
Study design for predicting dropout in chronic pain patients undergoing cannabis therapy.

Chronic pain, defined by the International Association for the Study of Pain (IASP) as an unpleasant sensory and emotional experience linked to actual or potential tissue damage (Raja et al., 2020), affects an estimated 30% of individuals globally, imposing profound personal and societal burdens (Dahlhamer et al., 2018). Unlike acute pain, which serves as a protective mechanism, chronic pain persists beyond 3 months, often becoming a disease (Treede et al., 2019). Traditional pharmacological treatments for chronic pain, such as opioids and NSAIDs, are usually inadequate due to incomplete pain relief, risks of dependency (Institute of Medicine, Board on Health Sciences Policy, Committee on Advancing Pain Research, Care, and Education, 2011), and adverse side effects. These limitations have driven increasing interest in alternative therapies, including medical cannabis, which offers potential analgesic and anti-inflammatory benefits (Vučković et al., 2018). Despite its growing use, the clinical application of cannabis remains hindered by substantial inter-individual variability in outcomes (Wang et al., 2023; Tait et al., 2023). This variability underscores the challenges posed by the pharmacokinetics and pharmacodynamics of cannabis, which are shaped by its rich phyto-complex. Cannabis contains over 100 cannabinoids distributed across 18 chemical classes, including terpenes, flavonoids, and alkaloids, with their concentrations influenced by factors such as extraction methods and product brands (Foster et al., 2019). Once absorbed, cannabinoids undergo extensive hydroxylation by cytochrome P450 enzymes, followed by glucuronidation and excretion (Chayasirisobhon, 2019) before their active forms bind to CB1 and CB2 receptors to mediate pharmacological effects. Furthermore, these cannabinoids interact with other compounds in the phyto-complex, resulting in potential synergistic and entourage impact (Anand et al., 2021). This complexity and individual biological diversity contribute to the wide variability in clinical outcomes. Pharmacogenetics, the study of how genetic differences influence drug response, has emerged as a promising tool for understanding and predicting patient-specific outcomes in cannabis therapy (Papastergiou et al., 2020). Research has identified polymorphisms in genes such as ABCB1, TRPV1, and UGT2B7 as potential determinants of cannabis efficacy and tolerability (Poli et al., 2022). These genetic markers could serve as predictors for identifying patients who are more likely to benefit from cannabis therapy or who are at risk of dropping out due to poor outcomes or adverse effects. While pharmacogenetics holds promise for personalizing cannabis therapy, translating genetic insights into clinical practice requires robust analytical methods capable of handling complex and multidimensional data. Machine learning (ML) has emerged as a transformative tool in this context, offering advanced capabilities to analyze large datasets and uncover patterns that might elude traditional statistical approaches (Delgado et al., 2018). ML models can integrate genetic, clinical, and pharmacological data to predict patient-specific outcomes, optimize therapeutic strategies, and ultimately improve the quality of care (Frusciante et al., 2022; Guerranti et al., 2021). In the context of chronic pain management, ML has the potential to identify key predictors of therapy success and dropout, facilitating a more targeted and efficient approach to treatment (Visibelli et al., 2023). Despite the potential of ML in healthcare, there is still a critical gap in its application to predicting dropout outcomes in chronic pain patients undergoing cannabis therapy. Most existing studies concentrate on treatment efficacy or side effect profiles (McMahon et al., 2023) without addressing the factors that drive patients to discontinue therapy. Understanding and mitigating dropout is crucial not only for enhancing patient outcomes but also for optimizing the allocation of healthcare resources and advancing precision medicine. In this study, we aim to address this gap by developing an ML model to predict therapy dropout in chronic pain patients treated with cannabis. Using a dataset comprising genetic, clinical, and pharmacological information, we investigate the interplay between pharmacogenetics and treatment outcomes to identify key predictors of dropout. This research seeks to advance our understanding of patient retention in cannabis therapy and to pave the way for personalized interventions that improve treatment adherence and efficacy. The workflow of this study is summarized in Figure 1.

## 2 Materials and methods

### 2.1 Data source

Between November 2018 and September 2020, 565 Caucasian patients suffering from chronic pain and with inadequate response to standard therapies were enrolled in a study conducted by Azienda USL Toscana Sud-Est at San Donato Hospital (Department of Pain Medicine and Palliative Care, Arezzo, Italy), as described in previous work (Poli et al., 2022). Participants provided written consent for their genotyping and therapeutic cannabis treatment. The study was approved by the Tuscan Regional Ethical Committee (No. 1287) on May 15, 2018, the study adhered to the 2008 revision of the Helsinki Declaration. The study design included an initial visit for diagnosis and prescription of medical cannabis, followed by four quarterly follow-ups to adjust therapy based on patient responses. The cannabis preparations used were derived from multiple varieties, each with specific THC and CBD ratios. The initial dose of THC prescribed was standardized at 5 mg per day, regardless of the cannabis variety, and extraction was performed according to the SIFAP (Italian Association of Compound Pharmacists) protocol under the regulations of the Italian Ministry of Health. During the visits, pain intensity was assessed using the Visual Analogue Scale (VAS) (Begum and Hossain, 2019), which rates pain intensity from 0 (indicating no pain) to 10 (indicating the worst pain). Based on prior studies, VAS scores were divided as <3.4 for mild pain, 3.5–7.4 for moderate pain, and >7.5 for severe pain in patients with chronic musculoskeletal pain (Boonstra et al., 2014). Additionally, the Hospital Anxiety and Depression Scale (HADS) (Zigmond and Snaith, 1983) was used to monitor psychological well-being. Patients who discontinued treatment were also recorded, allowing detailed tracking of therapeutic response and tolerability across the cohort. In addition, patients were genotyped for eight polymorphisms associated with drug metabolism, opioid pharmacology, and pain perception, based on previous research (Poli et al., 2022) linking these genes to cannabis effects. The genes selected were MDR1/ABCB1 (rs1045642), TRPV1 (rs8065080), UGT2B7 (rs7438135), CYP3A4 (rs2242480), CNR1 (rs1049353), COMT (rs4680), FAAH (rs2295632), and CYP3A4 (rs35599367).

### 2.2 Integrated clinical and treatment dataset

A comprehensive dataset of 542 anonymized individuals, each assigned a unique secure identifier, was developed to provide an in-depth patient profile by integrating clinical, genetic, and pharmacological data. Each patient is characterized by two primary categories of information: clinical characteristics and treatment-related information. The clinical profile encompasses variables such as age (ranging between 10 and 97 years), gender (male/female), the specific pathology diagnosed (e.g., central nervous system disorders, rheumatoid arthritis, inflammatory conditions, neuropathic pain, and others), as well as genetic polymorphism data. Meanwhile, the treatment-related information is split into two-time points: baseline and the final follow-up. Baseline data include initial daily doses of CBD and THC (measured in milligrams), binary indicators for the use of painkillers or other medications (yes/no), scores on the Visual Analog Scale (VAS, 0–10), assessments from the Hospital Anxiety and Depression Scale (HADS), reported therapeutic benefits, and the presence of side effects (yes/no). At the last follow-up, recorded parameters include updated daily doses of CBD and THC (in milligrams) and VAS scores (0–10). Additionally, the dataset features a “Drop” variable, a binary marker identifying patients who stopped treatment early. The pre-processed dataset encodes each categorical value between 0 and n classes-1, while the age column was standardized with a mean of 0 and a standard deviation (SD) of 1 due to its significantly higher range than the other values.

### 2.3 Data preprocessing

In this study, we applied a data preprocessing pipeline to ensure the dataset was clean, standardized, and suitable for analysis. All categorical variables were numerically encoded between 0 and n classes-1 to be compatible with the ML model. The age column was standardized by subtracting the mean and dividing by the standard deviation to ensure that features with different scales did not disproportionately influence the model. Missing data was managed systematically to maintain the integrity and reliability of the dataset. For numerical features, we employed the median to minimize the influence of outliers. Categorical variables were imputed using the mode to ensure consistency with the most frequently observed category. The decision to impute missing values rather than discard incomplete records was driven by the need to preserve valuable information, especially given the complexity of the dataset.

### 2.4 Machine learning method

To develop an ML model for predicting therapy dropout in chronic pain patients treated with cannabis, we selected the random forest (RF) (Breiman, 2001) model as the most effective classifier. This choice was based on RF’s capability to handle complex datasets and its robustness against overfitting. RF is a powerful ensemble learning method that combines multiple decision trees, each trained on a random subset of the data, to improve overall prediction accuracy. In addition to RF, we tested other machine learning models, including Logistic Regression, Support Vector Machine, and eXtreme Gradient Boosting. However, RF consistently outperformed these alternatives. Since the primary goal of this study was to identify the factors influencing therapy dropout, we present only the results from the RF model, which provided the most effective approach. Hyperparameter tuning was conducted using GridSearchCV, systematically exploring combinations of parameters to balance model complexity and computational efficiency while ensuring robust performance. The optimized model configuration consisted of 100 trees, a maximum depth of 40, and a minimum of two samples per split and leaf node. Class weights were adjusted to address data imbalance. Model performance was primarily evaluated using accuracy, defined as the ratio of correct predictions to the total number of predictions, providing an overall measure of model effectiveness. In addition, the receiver operating characteristic (ROC) curve was used to assess the trade-off between true and false positive rates. The area under the ROC curve (AUC) was also calculated, with higher AUC values indicating better model performance in distinguishing between the two classes. Feature selection was not explicitly performed, as the data had already been carefully curated to comprehensively represent the trial population. Furthermore, the RF model inherently performs feature selection by selecting a random subset of features at each tree split, ensuring that only the most relevant features are considered for each decision tree.

### 2.5 Descriptors analysis

We also report the contribution of the eight key features in the prediction through the SHapley Additive exPlanations (SHAP) technique (Hartono et al., 2020). SHAP methods assign a score to each input feature based on its influence on the target variable. In our context, SHAP values highlight the significance of each attribute by quantifying its contribution to the dropout prediction, with higher values indicating attributes essential for the decision-making processes. SHAP values are based on Shapley values, a concept from cooperative game theory that attributes contributions to individual players within a game. In the context of ML, SHAP values assign each feature an importance score for a specific prediction, offering insights into how each feature impacts the model’s output.

## 3 Results

### 3.1 Random forest prediction

**Figure 2:**
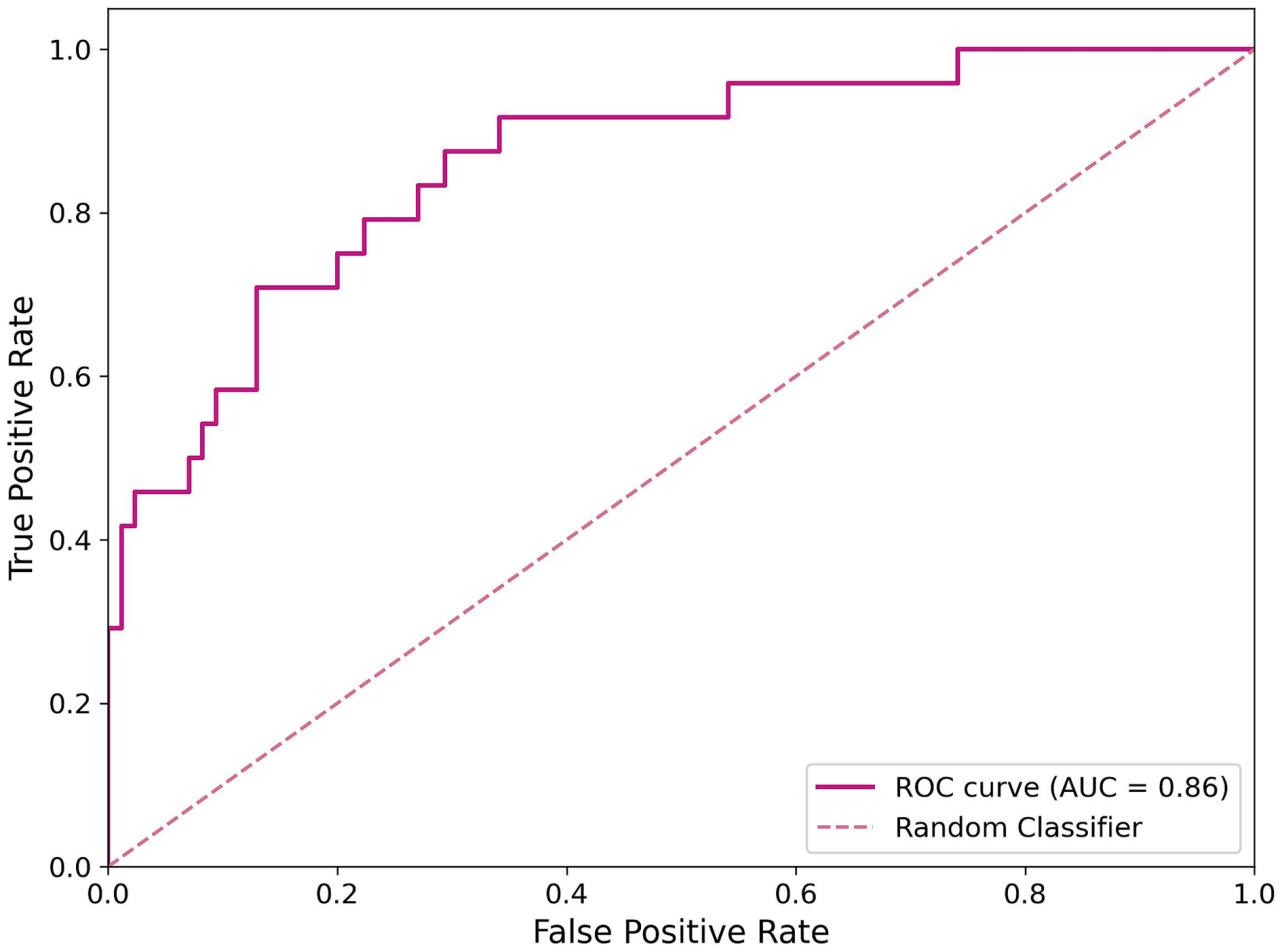
ROC curve of the classification model. The magenta line represents the RF best model’s performance, and the dashed line indicates a random classifier.

The pre-processed dataset was divided into a training set (80%) and a test set (20%). The training set was used to make the model learn the hidden features while the test set evaluated the model’s performance after training. To optimize the model’s hyperparameters, a GridSearch procedure was performed, systematically searching through a grid of hyperparameter values to identify the best combination that maximizes model performance. We implemented a model with 100 trees with a maximum depth of 40, and it requires at least two samples per leaf and two samples for splitting internal nodes. Moreover, the model balances class weights to handle class imbalance. Training and testing were performed over 1,000 iterations, using a unique dataset split. The model achieved a mean accuracy of 0.80, with an SD of 0.021 and a maximum accuracy of 0.86. The ROC curve illustrating the best model performance is shown in Figure 2, with an AUC value of 0.86.

### 3.2 Predictor importance assessment via SHAP

**Figure 3:**
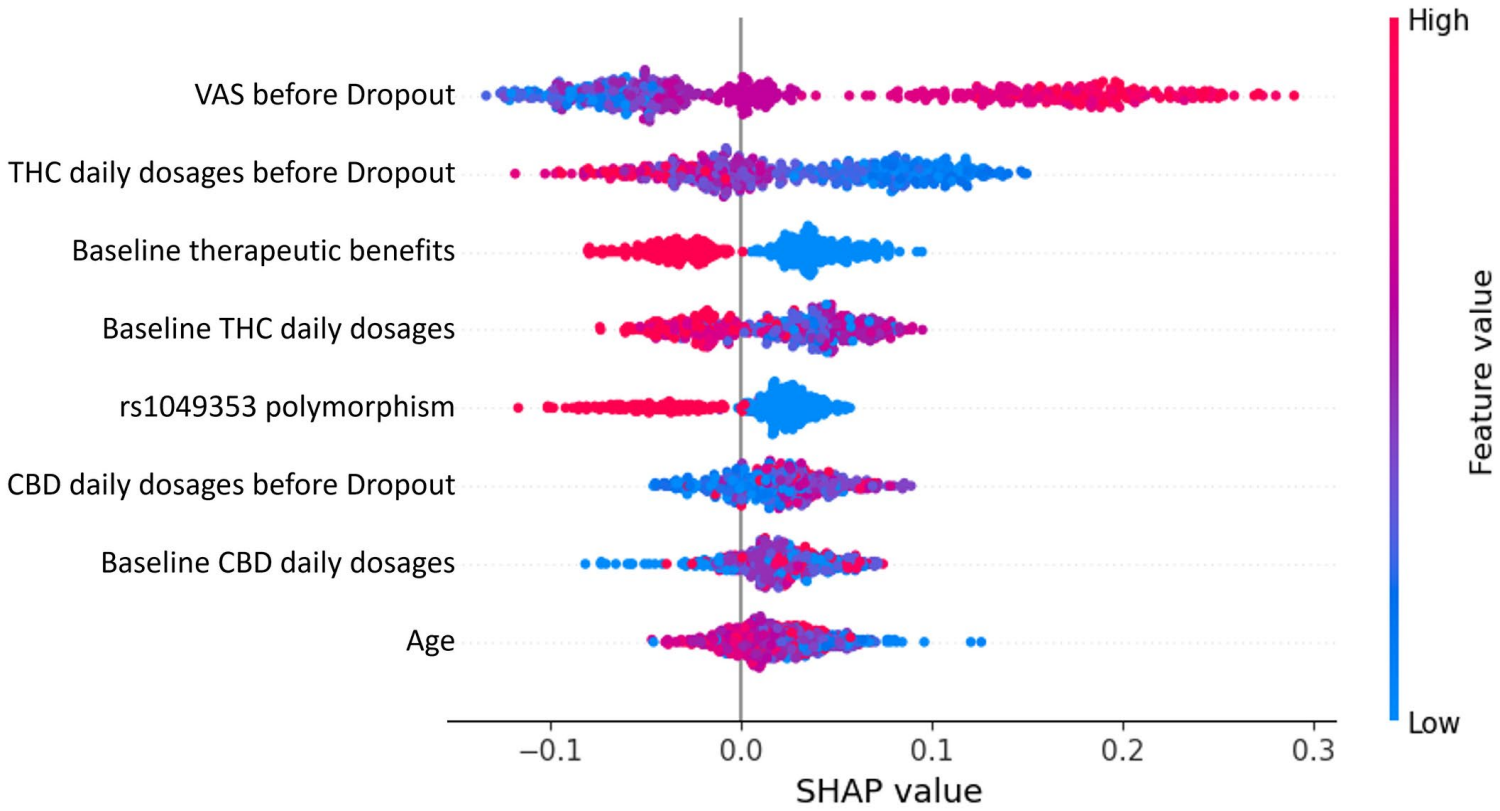
SHAP summary plot depicting the eight most important features influencing therapy dropout predictions in chronic pain patients treated with cannabis. Each dot represents a patient’s data, with the *x*-axis indicating the SHAP value (feature contribution to dropout prediction). Colors represent feature values, transitioning from low (blue) to high (red).

To gain insights into the key factors influencing therapy dropout in chronic pain patients treated with cannabis, SHAP analysis was employed. The SHAP summary plot in Figure 3 provides an overview of the influence of the eight most important features on the prediction outcome.

Each dot in the SHAP summary plot represents a patient’s data for a specific feature. The *x*-axis position reflects the SHAP value, indicating the magnitude and direction of the feature’s contribution to the prediction. Positive SHAP values drive predictions toward dropout, while negative values push predictions away from dropout. Features are arranged on the *y*-axis in descending order of importance, with the most impactful features displayed at the top. Dot colors represent the feature values for individual patients, ranging from low values in blue to high values in red. Both numerical and categorical features use the same color gradient. However, for categorical features, a higher encoded value does not imply a hierarchical relationship but rather signifies a distinct category compared to a lower value.

The most influential feature, the final VAS, indicates that higher reported pain levels are strongly associated with an increased dropout probability. This is because patients who perceive inadequate pain relief are more likely to discontinue therapy. Similarly, THC daily dosages at the final and baseline stages significantly affect dropout predictions. Higher THC dosages are linked to increased probabilities of dropout, likely due to side effects such as cognitive impairment or anxiety outweighing therapeutic benefits. Conversely, baseline therapeutic benefits suggest that patients reporting greater initial benefits are less likely to drop out, highlighting the importance of early positive outcomes in maintaining adherence. The genetic rs1049353 polymorphism is an important factor in the analysis. In this context, the SHAP plot highlights how different genotypes influence the prediction outcome. The red dots correspond to individuals with the CT genotype, while the blue dots represent those with the CC genotype. The TT genotype, however, is underrepresented in the sample, meaning there are too few individuals with this genotype to draw meaningful conclusions. As a result, the TT genotype is excluded from the analysis to avoid biased interpretations or unreliable results. Additionally, age and CBD daily dosages at the final and baseline stages contribute to shaping predictions, though less significantly. Lower CBD dosages tend to slightly increase the likelihood of dropout, whereas higher CBD dosages appear to moderate THC-related side effects, promoting adherence.

## 4 Discussion

The ML model developed in this study demonstrated strong predictive performance, achieving a mean accuracy of 80% and an AUC of 0.86. These metrics highlight the robustness of the RF classifier in identifying key predictors of therapy dropout among chronic pain patients treated with cannabis. To enhance interpretability, SHAP analysis was employed to determine the relative importance of various features, providing deeper insights into the factors driving the model’s predictions. Visual Analog Scale (VAS) scores emerged as a critical predictor of treatment trajectory. A decreasing VAS trend correlated strongly with reduced therapy dropout rates and higher patient satisfaction, while minimal changes indicated suboptimal pain relief (Giorgi et al., 2020; Wang et al., 2021; Harris et al., 2022). Beyond dropout prediction, VAS scores provided valuable insights into cannabis’s broader impact on clinical parameters, including sleep quality, fatigue, and anxiety (Bapir et al., 2023; Cahill et al., 2021). The variability in outcomes emphasizes the importance of considering individual patient profiles, including comorbidities such as anxiety or depression (Romero-Sandoval et al., 2018). The SHAP analysis identified cannabinoid dosing patterns as significant predictors of therapy adherence, with baseline THC levels showing greater impact on model predictions than final levels. CBD demonstrated a stabilizing influence throughout therapy, supporting its role in moderating THC-related side effects such as anxiety and cognitive impairment (Foster et al., 2019; Chayasirisobhon, 2019; Anand et al., 2021). The interplay between these cannabinoids is influenced by individual variability in pharmacokinetics and pharmacodynamics, affected by genetic polymorphisms and prior cannabis exposure (Babayeva and Loewy, 2023). Genetic analysis revealed rs1049353, a variant in the CNR1 gene encoding the cannabinoid receptor type 1 (CB1), as a key predictor of therapy dropout. Patients carrying the CT genotype showed significantly higher discontinuation rates compared to those with the CC genotype, likely due to altered CB1 receptor sensitivity or intracellular signaling pathways crucial for THC’s psychoactive and analgesic effects (Poli et al., 2022). This polymorphism has broader implications beyond cannabis therapy, having been associated with substance dependency (Pabalan et al., 2021; Zhang et al., 2004) and cognitive/psychiatric side effects during long-term cannabis treatment (Zeraatkar et al., 2022). These findings suggest a structured approach to optimizing cannabis-based pain management. Initial genetic screening for the rs1049353 variant should inform dosing strategies: CT genotype patients should start at 2.5 mg THC/day (versus standard 5 mg/day), combined with higher CBD ratios (2:1 CBD: THC) to minimize side effects. VAS scores should be monitored weekly during the first month, then biweekly for the next 2 months. A less than 30% improvement in VAS scores by week 4 should trigger a comprehensive treatment review, including dosing adjustments and assessment of concurrent symptoms. For patients showing minimal VAS improvement (<15%) despite dose optimization, early intervention with complementary pain management strategies could help prevent dropout. THC dose escalation should proceed more cautiously in CT genotype patients, with increases limited to 1.25 mg/week compared to the standard 2.5 mg/week protocol. Implementation of these monitoring protocols could be facilitated through mobile health applications, enabling real-time symptom tracking and automated alert systems for concerning trends in VAS scores or side effect reports. Several limitations should be considered when interpreting these results. The study’s focus on Caucasian patients may limit generalizability to other populations. Environmental and psychosocial factors, such as socioeconomic status and healthcare access, were not included despite their potential influence on therapy adherence. Additionally, while cannabis products are available in various formulations and routes of administration (Lucas et al., 2018), this information was excluded from our analysis. Given that pharmacokinetics and side effects depend heavily on administration methods and compound formulations, future research should evaluate long-term tolerability, functional outcomes, and alternate delivery routes. External validation represents a crucial next step to ensure model robustness and generalizability. Future efforts should focus on testing the model with independent datasets from diverse clinical settings and populations. This could involve multi-institutional collaboration to access comparable genetic, clinical, and pharmacological data. Practical implementation could include developing a decision support system that integrates genetic and clinical profiles to assess dropout risk and guide therapeutic strategies. Integration with electronic health records could streamline decision-making, improving both patient care and resource efficiency. Initial pilot studies and clinician training would ensure effective implementation and demonstrate the potential of combining machine learning and pharmacogenetics in personalized chronic pain management.

## 5 Conclusion

This study highlights the potential of ML in predicting therapy dropout among chronic pain patients undergoing cannabis treatment. By incorporating clinical, pharmacological, and genetic data, we identified key factors that play a crucial role in patient adherence. The SHAP analysis provided a detailed perspective on the complex interactions between these variables, emphasizing the value of personalized approaches in cannabis-based therapies. However, a key limitation of this study is that our dataset included only Caucasian patients, which may limit the generalizability of our findings to other ethnic populations. Given that genetic variations across ethnic groups can influence drug metabolism and therapeutic responses, our results—particularly those related to genetic polymorphisms like rs1049353—may not be directly applicable to non-Caucasian populations. Furthermore, cultural differences in pain perception, treatment preferences, and healthcare-seeking behaviors could impact therapy adherence patterns. Future studies should include more ethnically diverse cohorts to validate these findings and explore potential variations in cannabis treatment outcomes across different populations. Ultimately, this research demonstrates how integrating ML and pharmacogenetics can drive precision medicine in chronic pain management, advancing tailored interventions that improve patient outcomes.

## Data Availability

The data analyzed in this study is subject to the following licenses/restrictions: the data presented in this study are available on request from the corresponding author. The data are not publicly available because we are evaluating an eligible publicly accessible repository. Requests to access these datasets should be directed to anna.visibelli2@unisi.it.

## References

[ref1] Anand U. Pacchetti B. Anand P. Sodergren M. H. (2021). Cannabis-based medicines and pain: a review of potential synergistic and entourage effects. Pain Manag. 11, 395–403. doi: 10.2217/pmt-2020-0110 33703917

[ref2] Babayeva M. Loewy Z. G. (2023). Cannabis pharmacogenomics: a path to personalized medicine. Curr. Issues Mol. Biol. 45, 3479–3514. doi: 10.3390/cimb45040228, PMID: 37185752 PMC10137111

[ref3] Bapir L. Erridge S. Nicholas M. Pillai M. Dalavaye N. Holvey C.. (2023). Comparing the effects of medical cannabis for chronic pain patients with and without co-morbid anxiety: a cohort study. Expert. Rev. Neurother. 23, 281–295. doi: 10.1080/14737175.2023.2181696, PMID: 36803620

[ref4] Begum M. R. Hossain M. A. (2019). Validity and reliability of Visual Analogue Scale (VAS) for pain measurement. J. Med. Case Rep. Rev. 2:2.

[ref5] Boonstra A. M. Schiphorst Preuper H. R. Balk G. A. Stewart R. E. (2014). Cut-off points for mild, moderate, and severe pain on the Visual Analogue Scale for pain in patients with chronic musculoskeletal pain. Pain 155, 2545–2550. doi: 10.1016/j.pain.2014.09.014, PMID: 25239073

[ref6] Breiman L. (2001). Random forests. Mach. Learn. 45, 5–32. doi: 10.1023/A:1010933404324

[ref8] Cahill S. P. Lunn S. E. Diaz P. Page J. E. (2021). Evaluation of patient reported safety and efficacy of cannabis from a survey of medical cannabis patients in Canada. Front. Public Health 9:626853. doi: 10.3389/fpubh.2021.626853, PMID: 34095048 PMC8172603

[ref9] Chayasirisobhon S. (2019). Cannabis and neuropsychiatric disorders: an updated review. Acta Neurol. Taiwan 28, 27–39. 31867704

[ref10] Dahlhamer J. Lucas J. Zelaya C. Nahin R. Mackey S. DeBar L.. (2018). Prevalence of chronic pain and high-impact chronic pain among adults—United States, 2016. MMWR Morb. Mortal Wkly. Rep. 67, 1001–1006. doi: 10.15585/mmwr.mm6736a2, PMID: 30212442 PMC6146950

[ref11] Delgado D. A. Lambert B. S. Boutris N. McCulloch P. C. Robbins A. B. Moreno M. R.. (2018). Validation of Digital Visual Analog Scale pain scoring with a traditional paper-based visual analog scale in adults. J. Am. Acad. Orthop. Surg. Glob. Res. Rev. 2:e088. doi: 10.5435/JAAOSGlobal-D-17-00088, PMID: 30211382 PMC6132313

[ref12] Foster B. C. Abramovici H. Harris C. S. (2019). Cannabis and cannabinoids: kinetics and interactions. Am. J. Med. 132, 1266–1270. doi: 10.1016/j.amjmed.2019.05.017, PMID: 31152723

[ref14] Frusciante L. Visibelli A. Geminiani M. Santucci A. Spiga O. (2022). Artificial intelligence approaches in drug discovery: towards the laboratory of the future. Curr. Top. Med. Chem. 22, 2176–2189. doi: 10.2174/1568026622666221006140825, PMID: 36201265

[ref15] Giorgi V. Bongiovanni S. Atzeni F. Marotto D. Salaffi F. Sarzi-Puttini P. (2020). Adding medical cannabis to standard analgesic treatment for fibromyalgia: a prospective observational study. Clin. Exp. Rheumatol. 38, 53–59. 32116208

[ref17] Guerranti F. Mannino M. Baccini F. Bongini P. Pancino N. Visibelli A.. (2021). Caregiver matcher: graph neural networks for connecting caregivers of rare disease patients. Procedia Comput. Sci. 192, 1696–1704. doi: 10.1016/j.procs.2021.08.174

[ref18] Harris M. Erridge S. Ergisi M. Nimalan D. Kawka M. Salazar O.. (2022). UK Medical Cannabis Registry: an analysis of clinical outcomes of medicinal cannabis therapy for chronic pain conditions. Expert. Rev. Clin. Pharmacol. 15, 473–485. doi: 10.1080/17512433.2022.2017771 34937477

[ref19] Hartono N. T. P. Thapa J. Tiihonen A. Oviedo F. Batali C. Yoo J. J.. (2020). How machine learning can help select capping layers to suppress perovskite degradation. Nat. Commun. 11:11. doi: 10.1038/s41467-020-17945-4, PMID: 32820159 PMC7441172

[ref20] Institute of Medicine, Board on Health Sciences Policy, Committee on Advancing Pain Research, Care, and Education (2011). Relieving pain in America: a blueprint for transforming prevention, care, education, and research. Washington, DC: The National Academies Press. 22553896

[ref21] Lucas C. J. Galettis P. Schneider J. (2018). The pharmacokinetics and the pharmacodynamics of cannabinoids. Br. J. Clin. Pharmacol. 84, 2477–2482. doi: 10.1111/bcp.13710, PMID: 30001569 PMC6177698

[ref22] McMahon A. N. Varma D. S. Fechtel H. Sibille K. Li Z. Cook R. L.. (2023). Perceived effectiveness of medical cannabis among adults with chronic pain: findings from interview data in a three-month pilot study. Cannabis 6, 62–75. doi: 10.26828/cannabis/2023/000149, PMID: 37484052 PMC10361798

[ref23] Pabalan N. Chaweeborisuit P. Tharabenjasin P. Tasanarong A. Jarjanazi H. Eiamsitrakoon T.. (2021). Associations of CB1 cannabinoid receptor (CNR1) gene polymorphisms. Medicine 100:e27343. doi: 10.1097/MD.0000000000027343, PMID: 34713823 PMC8556036

[ref24] Papastergiou J. Li W. Sterling C. van den Bemt B. (2020). Pharmacogenetic-guided cannabis usage in the community pharmacy: evaluation of a pilot program. J. Cannabis Res. 2, 2522–5782. doi: 10.1186/s42238-020-00033-1, PMID: 33526106 PMC7819344

[ref25] Poli P. Peruzzi L. Maurizi P. Mencucci A. Scocca A. Carnevale S.. (2022). The pharmacogenetics of cannabis in the treatment of chronic pain. Genes 13:1832. doi: 10.3390/genes13101832, PMID: 36292717 PMC9601332

[ref26] Raja S. N. Carr D. B. Cohen M. Finnerup N. B. Flor H. Gibson S.. (2020). The revised International Association for the Study of Pain definition of pain: concepts, challenges, and compromises. Pain 161, 1976–1982. doi: 10.1097/j.pain.0000000000001939, PMID: 32694387 PMC7680716

[ref27] Romero-Sandoval E. A. Fincham J. E. Kolano A. L. Sharpe B. N. Alvarado-Vázquez P. A. (2018). Cannabis for chronic pain: challenges and considerations. Pharmacotherapy 38, 651–662. doi: 10.1002/phar.2115, PMID: 29637590

[ref28] Tait J. Erridge S. Holvey C. Coomber R. Usmani A. Sajad M.. (2023). Clinical outcome data of chronic pain patients treated with cannabis-based oils and dried flower from the UK Medical Cannabis Registry. Expert. Rev. Neurother. 23, 413–423. doi: 10.1080/14737175.2023.2195551, PMID: 37021592

[ref29] Treede R. D. Rief W. Barke A. Aziz Q. Bennett M. I. Benoliel R.. (2019). Chronic pain as a symptom or a disease: the IASP Classification of Chronic Pain for the International Classification of Diseases (ICD-11). Pain 160, 19–27. doi: 10.1097/j.pain.0000000000001384, PMID: 30586067

[ref30] Visibelli A. Peruzzi L. Poli P. Scocca A. Carnevale S. Spiga O.. (2023). Supporting machine learning model in the treatment of chronic pain. Biomedicines 11:1776. doi: 10.3390/biomedicines11071776, PMID: 37509416 PMC10376077

[ref31] Vučković S. Srebro D. Vujović K. S. Vučetić Č. Prostran M. (2018). Cannabinoids and pain: new insights from old molecules. Front. Pharmacol. 9:1259. doi: 10.3389/fphar.2018.01259, PMID: 30542280 PMC6277878

[ref32] Wang C. Erridge S. Holvey C. Coomber R. Usmani A. Sajad M.. (2023). Assessment of clinical outcomes in patients with fibromyalgia: analysis from the UK Medical Cannabis Registry. Brain Behav. 13:e3072. doi: 10.1002/brb3.3072, PMID: 37199833 PMC10338741

[ref33] Wang L. Hong P. J. May C. Rehman Y. Oparin Y. Hong C. J.. (2021). Medical cannabis or cannabinoids for chronic non-cancer and cancer-related pain: a systematic review and meta-analysis of randomised clinical trials. BMJ 374:n1034. doi: 10.1136/bmj.n1034 34497047

[ref34] Zeraatkar D. Cooper M. A. Agarwal A. Vernooij R. W. M. Leung G. Loniewski K.. (2022). Long-term and serious harms of medical cannabis and cannabinoids for chronic pain: a systematic review of non-randomised studies. BMJ Open 12:e054282. doi: 10.1136/bmjopen-2021-054282, PMID: 35926992 PMC9358949

[ref35] Zhang P. W. Ishiguro H. Ohtsuki T. Hess J. Carillo F. Walther D.. (2004). Human cannabinoid receptor 1: 5′ exons, candidate regulatory regions, polymorphisms, haplotypes and association with polysubstance abuse. Mol. Psychiatry 9, 916–931. doi: 10.1038/sj.mp.4001560, PMID: 15289816

[ref36] Zigmond A. S. Snaith R. P. (1983). The Hospital Anxiety and Depression Scale. Acta Psychiatr. Scand. 67, 361–370. doi: 10.1111/j.1600-0447.1983.tb09716.x 6880820

